# Youth Demographic Characteristics and Risk Perception of Using Alternative Tobacco Products: An Analysis of the 2014–2015 Canadian Student Tobacco, Alcohol, and Drugs Survey (CSTADS)

**DOI:** 10.1177/1179173X211058150

**Published:** 2021-11-22

**Authors:** Udoka Okpalauwaekwe, Chinenye Nmanma Nwoke, Jacinthe Messier

**Affiliations:** 1 Department of Academic Family Medicine, College of Medicine, 7235 University of Saskatchewan, Saskatoon, SK, Canada; 2 Faculty of Health Sciences, 4512 University of Lethbridge, Lethbridge, AB, Canada; 3 Independent Researcher in Indigenous Studies, Project Coordinator, Siksika First Nation Project, Calgary, AB, Canada

**Keywords:** Alternative tobacco products, e-cigarette, hookah, risk perception, Saskatchewan, Canada

## Abstract

**Background:**

There is a growing attraction by youth to alternative tobacco products (ATPs) such as e-cigarettes and hookahs. This study investigated risk perceptions and demographic characteristics associated with ATP use in grade 8–10 students.

**Methods:**

Data were drawn from the 2014/15 cycle of the CSTADS. The analytic sample included 1819 students from a total pool of 42 094 students who completed the survey. Logistic regression models were used to examine factors (demographic characteristics and risk perception) associated with ATP use in the past 30 days.

**Results:**

12% of students in grade 8–10 self-identified as having used ATPs in the past 30-days, with a majority of students in grade 10 (56%). Male students had higher odds of reporting ATP use when compared to females. Although a lesser proportion of Indigenous students reported ATP use in comparison to White students (31% vs 61%), Indigenous students were 2.42 (1.49, 3.93) times as likely to use ATPs as White students. Students who perceived smoking hookah once in a while as “no to slight risk” were 1.58 (1.09, 2.28) times more likely to report ATP use than students who perceived “moderate to great risk.” Also, students who perceived using e-cigarettes on a regular basis as “no to slight risk” were 2.21 (1.53, 3.21) times more likely to report ATP use as students who perceived “moderate-great risk.”

**Conclusion:**

A significant number of grade 8–10 students use ATPs, especially e-cigarettes, with the misconception of minimal health risks. There remains the need to do more to counteract the rise in social and epidemiological alternative tobacco use trends among the youth.

## Introduction

Tobacco use has become a global challenge, as consuming forms are becoming more sophisticated and the cultivated tobacco marketplace becoming increasingly diverse.^1,2^ According to a 2019 World Health Organization (WHO) report on the global tobacco epidemic, tobacco (*Nicotiana* genus) products are considered the leading cause of premature mortality and disease burden worldwide, resulting in approximately 7 million preventable deaths annually.^3^ Tobacco use remains a significant health risk factor for several diseases, including cancers, cardiovascular conditions, diabetes mellitus, and chronic respiratory ailments.^4,5^ Additionally, tobacco and tobacco products are harmful to those who smoke and those exposed to second-hand smoke (SHS) or environmental tobacco smokes (ETS).^6^ The World Health Organization recognizes these harms and the need to prevent significant health challenges from using tobacco and tobacco products from escalating in the future.^7^

Although the habit of cigarette-smoking appears seemingly on a decline among the youth population in general, the purchasing of nonconventional alternative tobacco products (ATPs), such as e-cigarettes, vaping devices, pipes, hookahs, cigars, cigarillos, flavored tobacco products (FTPs), and smokeless tobacco products (STPs) are on the rise globally, including in Canada.^5,8–11^ Research has indicated that tobacco products and their modern alternatives introduced during later childhood and early adolescence are strongly associated with a lower probability of quitting during an individual’s lifespan.^1,5,8,9,12^ This is why adult users of nicotine-containing tobacco are more likely to be addicted from their youth onwards, resulting in a normalized behavioral response to all other tobacco products, including ATPs.^1,9^

The overall prevalence rates of cigarette-smoking among the younger adult population in Canada have shown a downward trend, decreasing from 45% in 1981 to 13.8% in 2015.^13^ However, tobacco use remains significantly high among youth in Saskatchewan compared to other Canadian provinces.^13^ According to the 2012 Canadian Tobacco Use Monitoring Survey (CTUMS), 22.6% of Saskatchewan youth (aged 15 years and over) reported using nicotine-tobacco and other forms of ATPs compared to the 10.1% nationally.^13,14^ Comparably, a 2015 survey by the Saskatchewan Alliance for Youth and Community Well-being (SAYCW) showed that 22.8% of Saskatchewan teenagers between 15 and 19 years old use ATPs, compared to 14.6% nationwide.^15^ In the Canadian prairies, the vast growing and purchasing of ATPs, including nontraditional tobacco use or tobacco misuse by Indigenous adolescents (use of tobacco for recreational purposes), has been reported to influence high prevalence rates.^16,17^ Unfortunately, with companies targeting youth with the assortment of new, appealing, and readily accessible ATPs, studies have reported that youth are misguided about the health risks of ATPs.^1,18,19^ Thus, we designed this study to determine the associations between ATP use in the past 30 days, youth demographic characteristics, and risk perception in a representative sample of grade 8–10 students in Saskatchewan from the Canadian Student Tobacco, Alcohol and Drugs Survey (CSTADS) 2014/15 data.^20^

## Methods

### Data Source and Participants

The Canadian Student Tobacco, Alcohol, and Drugs Survey (CSTADS) is an ongoing biennial school-based survey administered to grade 6–12 students nationally and facilitated by the Propel Centre for Population Health Impact at the University of Waterloo.^20^ The objective of the CSTADS is to collect data on youth substance use/abuse and other areas identified by schools as priorities, such as bullying, mental health, and students’ connectedness or sense of belonging in their school environment.^21^ The survey uses a stratified single-stage cluster design, with strata based on smoking rates in health regions and the type of school. The 2014/2015 survey was implemented in schools between October 2014 and May 2015, replacing the Youth Smoking Survey (YSS) used in the years prior.^21^ For this study, data were drawn from the 2014/15 survey cycle of the CSTADS.^20^

The 2014/15 CSTADS involved a total of 42 094 respondents from grade 6 to 12 across 336 schools in Canada.^20^ All schools that participated in the survey received a $100 honorarium, a school-specific profile, and summaries of their survey results. In Saskatchewan, 4010 students participated in the 2014/15 survey cycle, with a student-level response rate of 79%. The current study was limited to Saskatchewan students in grade 8–10 (n = 1819). Survey weights were assigned to adjust for nonresponse and nonrandom sample selection of the responding sample.

Permission to use the 2014/15 CSTADS data was obtained through an application to the Propel Centre for Population Health Impact at the University of Waterloo.^20^

### Outcome Variable

The outcome variable for this study was alternative tobacco products (ATPs) used in the past 30 days, categorized as a binary variable. Alternative tobacco product use was derived from a combination of the following questions in the CSTADS asking: “In the last 30-days, did you use any of the following?—Little cigars or cigarillos (plain or flavored), cigars (not including little cigars or cigarillos, plain or flavored), roll-your-own cigarettes (tobacco only, in rolling papers), bidis (little cigarettes hand-rolled in leaves, tied with string ends, and may come in different flavors), smokeless tobacco (chewing tobacco, pinch, snuff, or snus), a water-pipe (hookah) to smoke shisha (herbal or tobacco), blunt wraps (a tube made of tobacco used to roll cigarette (tobacco), or e-cigarettes (electronic cigarettes). All the variables used to derive the outcome variable were dichotomous (Yes or No). Students who answered yes to any of these questions were classified as ATP users.

### Potential Correlates

Potential correlates included respondents’ demographics (i.e., grade = 8, 9, 10; sex = male, female; ethnicity = White, Indigenous, other), and perception of harm to using ATPs (i.e., risk of smoking hookah once in a while; risk of smoking hookah on a regular basis; risk of smoking e-cigarettes once in a while; risk of smoking e-cigarettes on a regular basis). Perceptions of risk from ATP use were assessed with the statement “how much do you think people risk harming themselves when they do each of the following activities?” with responses re-categorized to (1) no to slight risk and (2) moderate to great risk.

### Statistical Analysis

Sample characteristics were described with frequency and proportions. Associations between ATP use in the past 30 days (i.e., ATP use vs no ATP use) and sample characteristics were determined using χ-square tests. Significant variables from the tests of association were used to choose variables for the multiple regression analysis (i.e., sex, ethnicity, grade, perception of harm of hookah use (once in a while, on a regular basis), and perception of harm of e-cigarette use (once in a while, on a regular basis).

Multivariable logistic regression was performed to identify predictive variables significantly associated with ATP use in the past 30 days and presented as odds ratios and 95% confidence intervals (CI). Stepwise model building was utilized. At each step of the model building, the highest nonsignificant variable was removed until all variables were significant. Variables removed were also tested to determine if they were confounding variables. No confounders were found.

Survey weights (https://uwaterloo.ca/tobacco-use-canada/about/analysis) were included in the analysis to accommodate the survey design and non-response bias and ensure that the findings were representative of the grade 8–10 population in Saskatchewan. A *P*-value <.05 was considered statistically significant. All analyses were performed with Stata IC 16 statistical software package (College Station, Texas, Stata Corporation).^22^

## Results

Data from a sample of 1819 survey respondents were analyzed. Past-30-day ATP use was reported by 11.6% of participants (211/1819); the majority were male (64%), White (61%) and in 10th grade (51%) (see Table 1).

**Table 1.** Demographic characteristics of grades 8-10 students in Saskatchewan, with and without alternative tobacco products use in the past 30 days.

	Alternative Tobacco Products Use (past 30 days), N = 211	No Alternative Tobacco Products Use (past 30 days), N = 1608	P values
Sex, %			
Female	35.88	50.77	.002
Male	64.12	49.23	
Grade, %			
Eight	12.03	34.22	<.001
Nine	31.81	33.29	
Ten	56.16	32.49	
Ethnicity, %			
White	61.45	72.59	<.001
Indigenous	30.99	9.40	
Other	7.56	18.01	

Survey-set command was used to determine *P* values of weighted variables.

More ATP users than nonusers perceived no to slight risk of occasional use of hookah for both occasional (71% ATP users vs 51% nonATP users) and regular (46% ATP users vs 33% nonATP users) use. Similar trends were noticed for ATP users vs non-users for e-cigarettes for both occasional (90% ATP users vs 71% nonATP users) and regular (71% ATP users vs 46% nonATP users) use (see Table 2).

**Table 2.** Perception of harm characteristics of grades 8-10 students in Saskatchewan, with and without alternative tobacco products use in the past 30 days.

	Alternative Tobacco Products Use (past 30 days), N=211	No Alternative Tobacco Products Use (past 30 days), N=1608	P values
Smoke a tobacco water-pipe (hookah) once in a while			
No risk to slight risk	70.91	50.79	<.001
Moderate risk to great risk	29.09	49.21	
Smoke a tobacco water-pipe (hookah) on a regular basis			
No risk to slight risk	45.71	33.35	.005
Moderate risk to great risk	54.29	66.65	
Use an e-cigarette (electronic cigarette) once in a while			
No risk to slight risk	89.90	70.82	<.001
Moderate risk to great risk	10.10	29.18	
Use an e-cigarette (electronic cigarette) on a regular basis			
No risk to slight risk	71.10	46.52	<.001
Moderate risk to great risk	28.90	53.48	

Survey-set command was used to determine *P* values of weighted variables.

Table 3 presents the results of the multivariable logistic regression analyses. From the bivariate analyses, male respondents were twice as likely as females, tenth graders were 4 times as likely as eighth graders, and Indigenous students were more than twice as likely as White students to report past-30-day ATP use. In contrast, identifying as Black, Asian, or Hispanic was protective (see Table 3).

**Table 3.** Final model of factors associated with alternative tobacco products use among grades 8-10 students in Saskatchewan.

	Adjusted OR	95% CI	P value
Sex			
Female	Reference	Reference	
Male	2.00	1.44-2.77	<.001
Grade			<.001
Eight	Reference	Reference	
Nine	2.31	1.50-3.56	.48
Ten	4.17	2.77-6.26	<.001
Ethnicity			<.001
White	Reference	Reference	
Indigenous	2.42	1.49-3.93	<.001
Other	.51	.31-.85	<.001
Smoke a tobacco water-pipe (hookah) once in a while			
Moderate risk to great risk	Reference	Reference	
No risk to slight risk	1.58	1.09-2.28	.015
Use an e-cigarette (electronic cigarette) on a regular basis			
Moderate risk to great risk	Reference	Reference	
No risk to slight risk	2.21	1.53-3.21	<.001

CI, confidence interval.

Note: Model adjusted for perception of smoking hookah on a regular basis and use of an e-cigarette once in a while.

Assessing perception of harm variables, respondents who stated that smoking hookah once in a while was “no risk to slight risk” were one and one-half times more likely to report past-30-day ATP use compared to those who responded, “great risk to moderate risk.” Similarly, respondents who stated that use of an e-cigarette on a regular basis was “no risk to slight risk” were more than twice more likely to report past-30-day ATP use compared to those who responded, “great risk to moderate risk.” (see Table 3).

## Discussion

We investigated the demographic characteristics of Saskatchewan students in grade 8–10 that used ATPs and explored the perception of health risk associated with ATP use. Our results showed that a more students who used ATPs in the last 30 days perceived “no risk to slight risks” of using hookahs and/or e-cigarettes occasionally or on a regular basis compared with students who had not used ATPs in the past 30 days. These results are comparable to findings from several similar studies exploring risk or harm perception of hookahs, e-cigarettes, flavored cigarettes, water-pipes, and other ATP forms.^1,5,9,18,23–27^ The common justification provided in these studies for this misperception was the notion that e-cigarettes and other forms of ATPs were beneficial alternatives for personal tobacco cessation and harm reduction.^1,5,9,18,23,24,27^ A recent survey of over 7000 youth tobacco and poly-tobacco users in the US reported that youth perceived e-cigarettes to be more popular and safer than combustible tobacco because of the novel technology that gauges nicotine strength, with added healthy flavors, hence reduced chances for addictiveness.^26^ Another very recent study exploring risk perceptions of hookah use among 671 youth reported that 45% of them believed hookahs were less harmful because the water, molasses, and fruity flavors in hookahs filter harmful or toxic substances better than with regular filter cigarettes.^5^ As such, youth find ATPs such as e-cigarettes, hookahs, and flavored tobacco products more attractive alternatives to cigarette-smoking.^5^ Comparable studies have also implicated the several reasons for the growing youth attraction to ATPs to include the increasing normalization of ATP use on social media, the glamorizing marketing strategies targeted at youth, lower and/or affordable cost, the assortment of flavored e-juices, e-cigarette designs, and characteristics (e.g., shape, style, and volume) and easy access to ATPs and other illicit substances, in part due to the recent legalization of cannabis in Canada.^8,18,28–31^ These preferences are then rationalized by youth as tools to fit in with peers, recreational sport for showing off vaping tricks, enhancement tools for stress and anxiety relief, and status symbols as with cigars and cigarillos among adults.^24,27,32,33^ Notwithstanding, this begs the crucial need for refortified health literacy efforts and media campaigns focusing on the addictive nature of nicotine which is native in all ATP forms.

Our study also showed that Indigenous students were 2 times more likely to use ATP in the past 30 days compared with White students. Consistent with this are similar findings from recent studies on tobacco use among Indigenous youth.^2,16,17,19,34–38^ These studies cite the influence of peer pressure, lack of social and community support, a loss of cultural identity, the impacts of colonization, and the intergenerational trauma (brought about by the residential school systems and the sixties scoop in Canada) to be responsible for the high prevalences of tobacco misuse (i.e., recreational or nontraditional use of tobacco).^2,16,17,19,34–38^ The high prevalence rates of tobacco misuse by Indigenous youth have also been implicated in leveraging the overall high rates of tobacco use in Saskatchewan province.^39,40^ Historically, Indigenous communities’ relationship with tobacco before colonization was not for recreational use^38^ because various species of tobacco were considered sacred and used (inhaled or smudged) in small amounts, and for limited time periods for medicinal, spiritual, and socio-cultural purposes.^38,41^ For example, *Nicotiana tabacum* and *N*. *rustica* plants have been valued and revered for thousands of years among many Indigenous communities.^42,43^ However, there has been a vital transfer, since colonial times, to recreational use of nicotine-based products by Indigenous peoples,^27,29,32^ all of which have resulted in severe and associated health concerns, particularly for young adults.^5,16,17,44^ Therefore, a key consideration in intervention efforts with Indigenous youth and communities should be on the nontraditional use of tobacco and tobacco products and not the narrative that “all tobacco use is bad,” rather, should differentiate between traditional (sacred) and nontraditional (recreational) use. Engaging with the Elders and Knowledge Keepers in Indigenous communities can facilitate the appropriate dissemination of this knowledge and the co-creation of community-driven and context-specific mitigation strategies.^45^

Males and students in grade 10 were significantly more likely to use ATP in the past 30 days compared with their female and lower-grade counterparts. These findings are consistent with similar studies among youth and school graders ^8,11,35,46–48^; and might be explained using the psychological reactance theory (PRT).^42^ PRT states that proscribed youthful attitudes will motivate an individual to pursue a basic need for self-determination, as a means to self-preserve autonomy, especially at the beginning of adult life.^42^ It is, therefore, conceivable that with the present preponderance of antitobacco interventions, tobacco-control and nicotine-free campaigns in middle to high-grade schools in Canada,^49^ may have instigated youth to react against these contemporary efforts as a way to assert self-determination towards tobacco and ATP control.^42^ Additionally, the perception of harm using ATPs such as e-cigarettes and hookahs have been correlated with gender differences in research studies, as males tended to consider themselves more invulnerable to illnesses or injuries compared with females.^1,23,28,50,51^ This still calls for concern as it could indicate that the health promotion and prevention strategies provided to middle to high-grade students in the province may be failing or needs restrategization.^52^

Our study highlights the importance of investigating patterns of tobacco and ATP use (as well as other substances) by grades and among youth in general. We believe our findings can provide foundational or additive basis for developing health prevention and cessation programs for youth in grade schools in respective Canadian provinces. Although Canada has demonstrated positive strides in reducing tobacco consumption among adults in nonIndigenous and Indigenous communities, there remains a need for continued and strengthen efforts, especially among our vulnerable and impressionable youth population.^36,37^ Evidence have supported strategies like aggressive health and social media campaigns in grade schools, increasing cost and access to ATPs, tailored age-specific addiction and cessation programs in youth communities, and policy actions (e.g., to reduce legal limits for nicotine concentrations in ATPs and flavor bans to minimize attraction to e-cigarettes and ATPs) in the general population.^16,27,53–55^ However, research has shown that culturally adapted interventions can reduce tobacco misuse among Indigenous communities.^34,37,56,57^ This would involve less aggressive measures, more importantly, community engagement with elders, Knowledge Keepers, and Indigenous community members.^36,54^ This is because research with Indigenous communities must be reflective of their norms, cultural values, and customs, as dictated in the Tri-Council Policy Statement (TCPS) on Ethical conduct for research involving First Nations, Inuit, and Métis peoples.^45^ Using this approach facilitates the sustainability of community-driven strategies that promote wellness within the community.^45^

### Strengths and Limitations

Although these analyses of cross-sectional population-level data identified some associations, it is important to acknowledge that they cannot imply a cause-and-effect relationship between correlates and the outcomes of ATP use in the past 30 days. All statistics reported are estimates, and caution needs to be taken in their application and interpretation. The cross-sectional design of this study provides a snapshot of ATP use among grade 8–10 students in Saskatchewan at a specific time and space. Also, the time limitation on the use of ATPs in general, as represented within the previous 30 days usage, may have biased the volume or frequency of ATP use. Furthermore, limiting the research to youth only in schools could affect the external validity of the results, as it is plausible that the majority of youth that uses ATP may not be attending school or attending classes’ in-person. However, the investigation showed strengths in its large sample capacity, being a national model with an adequate representation from across the province of Saskatchewan.

## Conclusion

A significant number of Saskatchewan grade 8–10 school students use ATPs, especially e-cigarettes and hookahs. As they get to higher grades, youth, mainly male and indigenous youth, seem to have little to no knowledge about the health risks of using ATPs. Our research results highlight the need for continued health literacy efforts on tobacco and ATPs in grade schools and a collective action to engage Indigenous youth in health and wellness promotion strategies that integrate their Indigenous ways of knowing.
